# Methyl 2,2-dimeth­oxy-8-oxo-1-oxaspiro­[4.5]deca-6,9-diene-3-carboxyl­ate

**DOI:** 10.1107/S1600536812011737

**Published:** 2012-03-24

**Authors:** Yongbing Lou

**Affiliations:** a School of Chemistry and Chemical Engineering, Southeast University, Southeast University Road 2, Jiangning District, 211189 Nanjing, People’s Republic of China

## Abstract

In the title mol­ecule, C_13_H_16_O_6_, the cyclo­hexa-1,4-diene ring adopts a flat boat conformation (r.m.s. deviation from planarity = 0.060 Å) and the five-membered tetra­hydro­furan ring adopts an envelope conformation with the carboxyl group-substituted C atom as the flap. The dihedral angle at the spiro junction is 89.1 (1)°. In the crystal, mol­ecules are linked through weak C—H⋯O and van der Waals inter­actions.

## Related literature

For background to bioactive tetronic acid derivatives, see: Fischer *et al.* (1993 ▶); Bayer Aktiengesellschaft (1995) ▶.
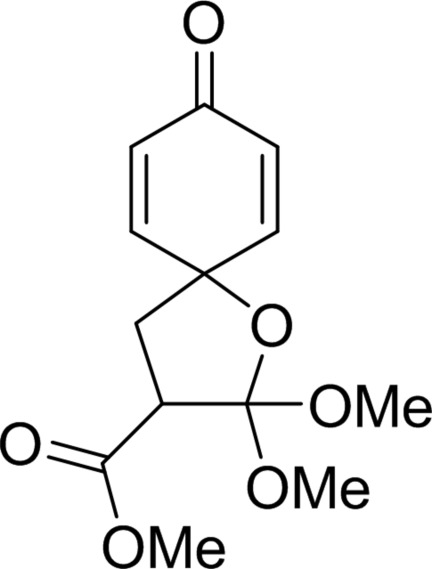


## Experimental

### 

#### Crystal data

C_13_H_16_O_6_
*M* *_r_* = 268.26
Monoclinic, 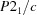

*a* = 6.5324 (7) Å
*b* = 11.7519 (12) Å
*c* = 17.4204 (18) Å
β = 97.723 (2)°
*V* = 1325.2 (2) Å^3^
*Z* = 4
Mo *K*α radiation
μ = 0.11 mm^−1^
*T* = 298 K
0.31 × 0.26 × 0.21 mm

#### Data collection

Bruker SMART CCD diffractometer
Absorption correction: multi-scan (*SADABS*; Bruker, 2000 ▶) *T*_min_ = 0.958, *T*_max_ = 0.978
7015 measured reflections
2588 independent reflections
2140 reflections with *I* > 2σ(*I*)
*R*_int_ = 0.020

#### Refinement

*R*[*F* ^2^ > 2σ(*F* ^2^)] = 0.048
*wR*(*F* ^2^) = 0.140
*S* = 1.05
2588 reflections
175 parameters
H-atom parameters constrained
Δρ_max_ = 0.29 e Å^−3^
Δρ_min_ = −0.22 e Å^−3^

### 

Data collection: *SMART* (Bruker, 2000 ▶); cell refinement: *SAINT* (Bruker, 2000 ▶); data reduction: *SAINT*; program(s) used to solve structure: *SHELXS97* (Sheldrick, 2008 ▶); program(s) used to refine structure: *SHELXL97* (Sheldrick, 2008 ▶); molecular graphics: *SHELXTL* (Sheldrick, 2008 ▶); software used to prepare material for publication: *SHELXTL*.

## Supplementary Material

Crystal structure: contains datablock(s) I, global. DOI: 10.1107/S1600536812011737/qk2030sup1.cif

Structure factors: contains datablock(s) I. DOI: 10.1107/S1600536812011737/qk2030Isup2.hkl

Supplementary material file. DOI: 10.1107/S1600536812011737/qk2030Isup3.cml

Additional supplementary materials:  crystallographic information; 3D view; checkCIF report

## Figures and Tables

**Table 1:** Hydrogen-bond geometry (Å, °)

*D*—H⋯*A*	*D*—H	H⋯*A*	*D*⋯*A*	*D*—H⋯*A*
C13—H13*A*⋯O3^i^	0.96	2.60	3.269 (3)	127

Symmetry code: (i) 
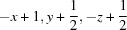
.

**Fig. 1.**
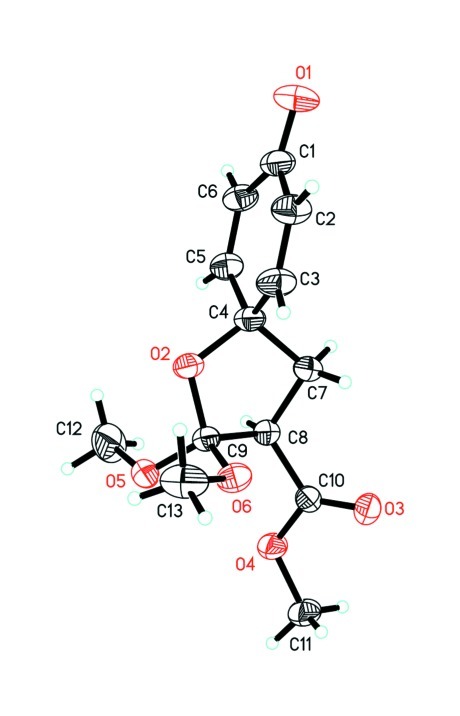
Molecular structure of the title compound. Displacement ellipsoids were drawn at the 30% probability level.

**Fig. 2.**
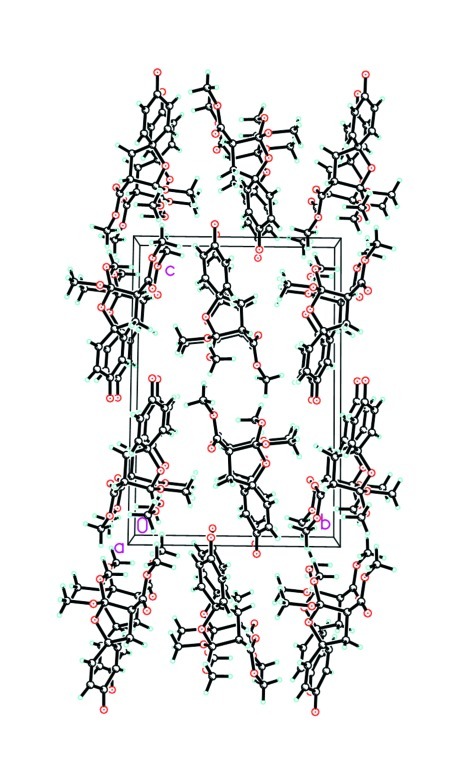
Packing diagram of the structure viewed down the a-axis.

**Fig. 3.**
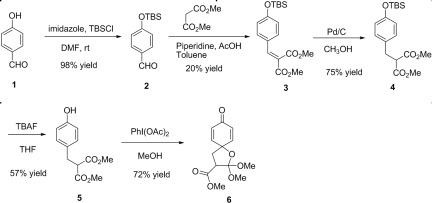
Synthetic route for the title compound.

## supplementary crystallographic information

### Comment

The chemistry of tetronic acid (tetrahydrofuran-2,4-dione) compounds has received increasing attention in recent years due to their high biological activity as herbicides and insecticides (Fischer *et al.*, 1993). The company Bayer AG has developed three tetronic acid pesticides, spirodiclofen, spiromesifen, and spirotetramat (Bayer Aktiengesellschaft, 1995), which are now in wide use in crop protection. As part of our studies in this area, we describe here the structure of the title compound (Scheme 1).

The title molecule (Fig. 1) contains one six-membered and one five-membered ring connected with a spiro-carbon C4. All bond lengths in this spiro system adopt normal values, e.g. the double bonds C2═C3, C5═C6, and C1═O1 with values of 1.320 (3) Å, 1.322 (3) Å, and 1.219 (2) Å, respectively. The cyclohexadienone unit is slightly bent to a flat boat conformation with atoms C2, C3, C5, C6 being practically coplanar and C1, C4, and O1 by 0.087 (3), 0.0163 (3), and 0.191 (5) Å, respectively, off from the plane of the former atoms. The five-membered tetrahydrofuran ring adopts an envelope conformation with C8 by 0.558 (3) Å out of the least-squares plane through O2, C4, C7, and C9 (their r.m.s. deviation from l.s. plane is 0.017 Å). In the crystal (Fig. 2), the molecules are linked through weak van der Waals and C-H···O interactions.

### Experimental

The starting material and all intermediates are known from literature and are obtained by standard procedures. The title compound was synthesized starting with 4-hydroxybenzaldehyde according to Fig. 3. using standard procedures for the intermediates 2 through 5. Then, to a solution of 5 (800 mg, 3.36 mmol) in MeOH (12 ml) was added a solution of PhI(OAc)_2_ (1.6 g, 4.97 mmol) in CH_2_Cl_2_ (7 ml) at room temperature. The mixture was stirred for 30 min. The solvent was removed under reduced pressure and the residue was purified by silica gel column chromatography (EtOAc: PE = 1:3) to afford 6 (648.1 mg, 72% yield). ^1^H NMR (400 MHz, CDCl_3_) *δ* 2.28 (dd, *J* =9.2 Hz, 13.6 Hz, 1H), 2.72 (dd, *J* = 8.0 Hz, 13.6 Hz, 1H), 3.38 (s, 3H), 3.46 (s, 3H), 3.78 (s, 3H), 6.15–6.20 (m, 2H), 6.89–6.92 (m, 1H), 7.07–7.10 (m, 1H). ^13^C NMR (100 MHz, CDCl_3_) *δ* 36.9, 48.4, 49.9, 51.1, 52.4, 75.8, 122.2, 127.6, 148.2, 149.2, 169.5, 185.0.

### Refinement

All H atoms were placed in calculated positions with C—H = 0.93–0.98Å and were included in the final cycle of refinement in the riding mode with *U*_iso_(H) = 1.2 or 1.5*U*_eq_(C).

### Crystal data

**Table:**

C_13_H_16_O_6_	*F*(000) = 568
*M**_r_* = 268.26	*D*_x_ = 1.345 Mg m^−^^3^
Monoclinic, *P*2_1_/*c*	Mo *K*α radiation, λ = 0.71073 Å
Hall symbol: -P 2ybc	Cell parameters from 2543 reflections
*a* = 6.5324 (7) Å	θ = 5.9–49.9°
*b* = 11.7519 (12) Å	µ = 0.11 mm^−^^1^
*c* = 17.4204 (18) Å	*T* = 298 K
β = 97.723 (2)°	Prismatic, colorless
*V* = 1325.2 (2) Å^3^	0.31 × 0.26 × 0.21 mm
*Z* = 4	

### Data collection

**Table:**

Bruker SMART CCD diffractometer	2588 independent reflections
Radiation source: fine-focus sealed tube	2140 reflections with *I* > 2σ(*I*)
Graphite monochromator	*R*_int_ = 0.020
φ and ω scans	θ_max_ = 26.0°, θ_min_ = 2.1°
Absorption correction: multi-scan (*SADABS*; Bruker, 2000)	*h* = −7→8
*T*_min_ = 0.958, *T*_max_ = 0.978	*k* = −14→14
7015 measured reflections	*l* = −13→21

### Refinement

**Table:**

Refinement on *F*^2^	Primary atom site location: structure-invariant direct methods
Least-squares matrix: full	Secondary atom site location: difference Fourier map
*R*[*F*^2^ > 2σ(*F*^2^)] = 0.048	Hydrogen site location: inferred from neighbouring sites
*wR*(*F*^2^) = 0.140	H-atom parameters constrained
*S* = 1.05	*w* = 1/[σ^2^(*F*_o_^2^) + (0.0751*P*)^2^ + 0.297*P*] where *P* = (*F*_o_^2^ + 2*F*_c_^2^)/3
2588 reflections	(Δ/σ)_max_ < 0.001
175 parameters	Δρ_max_ = 0.29 e Å^−^^3^
0 restraints	Δρ_min_ = −0.22 e Å^−^^3^

### Special details

**Table:**

Geometry. All e.s.d.'s (except the e.s.d. in the dihedral angle between two l.s. planes) are estimated using the full covariance matrix. The cell e.s.d.'s are taken into account individually in the estimation of e.s.d.'s in distances, angles and torsion angles; correlations between e.s.d.'s in cell parameters are only used when they are defined by crystal symmetry. An approximate (isotropic) treatment of cell e.s.d.'s is used for estimating e.s.d.'s involving l.s. planes.
Refinement. Refinement of *F*^2^ against ALL reflections. The weighted *R*-factor *wR* and goodness of fit *S* are based on *F*^2^, conventional *R*-factors *R* are based on *F*, with *F* set to zero for negative *F*^2^. The threshold expression of *F*^2^ > σ(*F*^2^) is used only for calculating *R*-factors(gt) *etc*. and is not relevant to the choice of reflections for refinement. *R*-factors based on *F*^2^ are statistically about twice as large as those based on *F*, and *R*- factors based on ALL data will be even larger.

### Fractional atomic coordinates and isotropic or equivalent isotropic displacement parameters (Å^2^)

**Table:**

	*x*	*y*	*z*	*U*_iso_*/*U*_eq_	
O1	−0.2524 (3)	0.60788 (16)	−0.03288 (8)	0.0876 (5)	
O2	0.0228 (2)	0.64745 (11)	0.26381 (7)	0.0560 (4)	
O3	0.5107 (3)	0.39189 (16)	0.31643 (8)	0.0826 (5)	
O4	0.34081 (19)	0.39820 (12)	0.41846 (7)	0.0571 (4)	
O5	0.08806 (19)	0.62430 (12)	0.39416 (7)	0.0567 (4)	
O6	0.35585 (19)	0.64129 (11)	0.32927 (7)	0.0546 (4)	
C1	−0.1786 (3)	0.60713 (17)	0.03521 (11)	0.0601 (5)	
C2	0.0197 (3)	0.66200 (19)	0.06170 (12)	0.0665 (6)	
H2	0.0846	0.7040	0.0267	0.080*	
C3	0.1080 (3)	0.65306 (19)	0.13416 (12)	0.0639 (6)	
H3	0.2302	0.6927	0.1491	0.077*	
C4	0.0216 (3)	0.58253 (16)	0.19334 (10)	0.0510 (4)	
C5	−0.1957 (3)	0.54671 (16)	0.16723 (11)	0.0553 (5)	
H5	−0.2722	0.5170	0.2039	0.066*	
C6	−0.2852 (3)	0.55504 (17)	0.09477 (12)	0.0601 (5)	
H6	−0.4186	0.5272	0.0815	0.072*	
C7	0.1620 (3)	0.47845 (17)	0.21615 (10)	0.0584 (5)	
H7A	0.1032	0.4096	0.1916	0.070*	
H7B	0.2989	0.4899	0.2018	0.070*	
C8	0.1695 (3)	0.47217 (14)	0.30329 (9)	0.0460 (4)	
H8	0.0463	0.4327	0.3160	0.055*	
C9	0.1579 (2)	0.59822 (14)	0.32513 (9)	0.0440 (4)	
C10	0.3594 (3)	0.41569 (15)	0.34472 (10)	0.0490 (4)	
C11	0.5178 (3)	0.35071 (19)	0.46569 (12)	0.0659 (6)	
H11A	0.5372	0.2734	0.4503	0.099*	
H11B	0.4961	0.3525	0.5191	0.099*	
H11C	0.6383	0.3945	0.4592	0.099*	
C12	−0.1168 (4)	0.5942 (3)	0.40212 (17)	0.0983 (10)	
H12A	−0.2091	0.6268	0.3604	0.147*	
H12B	−0.1506	0.6224	0.4506	0.147*	
H12C	−0.1304	0.5129	0.4008	0.147*	
C13	0.3702 (4)	0.76269 (19)	0.33775 (14)	0.0778 (7)	
H13A	0.3063	0.7985	0.2911	0.117*	
H13B	0.5130	0.7846	0.3475	0.117*	
H13C	0.3011	0.7861	0.3804	0.117*	

### Atomic displacement parameters (Å^2^)

**Table:**

	*U*^11^	*U*^22^	*U*^33^	*U*^12^	*U*^13^	*U*^23^
O1	0.0973 (12)	0.1070 (13)	0.0497 (9)	−0.0063 (10)	−0.0219 (8)	0.0074 (8)
O2	0.0571 (7)	0.0555 (7)	0.0502 (7)	0.0126 (6)	−0.0116 (6)	−0.0035 (5)
O3	0.0771 (10)	0.1179 (14)	0.0537 (9)	0.0494 (10)	0.0115 (7)	0.0034 (8)
O4	0.0570 (8)	0.0695 (9)	0.0441 (7)	0.0098 (6)	0.0041 (6)	0.0112 (6)
O5	0.0510 (7)	0.0703 (8)	0.0485 (7)	0.0051 (6)	0.0056 (6)	−0.0117 (6)
O6	0.0456 (7)	0.0566 (8)	0.0596 (8)	−0.0074 (5)	−0.0004 (6)	0.0070 (6)
C1	0.0633 (12)	0.0596 (11)	0.0518 (11)	0.0056 (9)	−0.0125 (9)	0.0026 (9)
C2	0.0652 (12)	0.0766 (14)	0.0554 (11)	−0.0081 (10)	−0.0006 (9)	0.0161 (10)
C3	0.0510 (11)	0.0787 (14)	0.0582 (12)	−0.0127 (9)	−0.0067 (9)	0.0101 (10)
C4	0.0487 (10)	0.0578 (11)	0.0431 (9)	0.0014 (8)	−0.0061 (7)	0.0020 (7)
C5	0.0511 (10)	0.0576 (11)	0.0547 (11)	−0.0061 (8)	−0.0021 (8)	0.0050 (8)
C6	0.0509 (10)	0.0617 (11)	0.0622 (12)	−0.0066 (9)	−0.0130 (9)	0.0016 (9)
C7	0.0630 (11)	0.0667 (12)	0.0420 (9)	0.0149 (9)	−0.0059 (8)	−0.0057 (8)
C8	0.0452 (9)	0.0484 (10)	0.0430 (9)	0.0024 (7)	0.0012 (7)	−0.0013 (7)
C9	0.0387 (8)	0.0505 (9)	0.0413 (9)	0.0014 (7)	−0.0001 (7)	−0.0002 (7)
C10	0.0560 (10)	0.0491 (9)	0.0412 (9)	0.0090 (8)	0.0039 (8)	−0.0024 (7)
C11	0.0665 (13)	0.0759 (14)	0.0525 (11)	0.0154 (10)	−0.0029 (9)	0.0168 (10)
C12	0.0590 (14)	0.152 (3)	0.0895 (18)	−0.0069 (15)	0.0299 (13)	−0.0282 (17)
C13	0.0921 (16)	0.0582 (12)	0.0756 (14)	−0.0239 (11)	−0.0158 (12)	0.0146 (11)

### Geometric parameters (Å, º)

**Table:**

O1—C1	1.219 (2)	C5—C6	1.321 (3)
O2—C9	1.4148 (19)	C5—H5	0.9300
O2—C4	1.444 (2)	C6—H6	0.9300
O3—C10	1.195 (2)	C7—C8	1.514 (2)
O4—C10	1.323 (2)	C7—H7A	0.9700
O4—C11	1.439 (2)	C7—H7B	0.9700
O5—C9	1.377 (2)	C8—C10	1.503 (2)
O5—C12	1.409 (3)	C8—C9	1.534 (2)
O6—C9	1.381 (2)	C8—H8	0.9800
O6—C13	1.436 (3)	C11—H11A	0.9600
C1—C6	1.460 (3)	C11—H11B	0.9600
C1—C2	1.465 (3)	C11—H11C	0.9600
C2—C3	1.320 (3)	C12—H12A	0.9600
C2—H2	0.9300	C12—H12B	0.9600
C3—C4	1.492 (3)	C12—H12C	0.9600
C3—H3	0.9300	C13—H13A	0.9600
C4—C5	1.492 (2)	C13—H13B	0.9600
C4—C7	1.549 (3)	C13—H13C	0.9600
			
C9—O2—C4	110.94 (13)	C10—C8—C9	111.85 (14)
C10—O4—C11	116.36 (15)	C7—C8—C9	101.87 (14)
C9—O5—C12	117.44 (16)	C10—C8—H8	109.4
C9—O6—C13	114.67 (16)	C7—C8—H8	109.4
O1—C1—C6	122.06 (19)	C9—C8—H8	109.4
O1—C1—C2	121.4 (2)	O5—C9—O6	106.90 (13)
C6—C1—C2	116.54 (16)	O5—C9—O2	108.78 (13)
C3—C2—C1	121.39 (19)	O6—C9—O2	111.98 (14)
C3—C2—H2	119.3	O5—C9—C8	117.70 (15)
C1—C2—H2	119.3	O6—C9—C8	106.86 (14)
C2—C3—C4	123.21 (18)	O2—C9—C8	104.72 (13)
C2—C3—H3	118.4	O3—C10—O4	123.58 (16)
C4—C3—H3	118.4	O3—C10—C8	125.45 (16)
O2—C4—C5	107.74 (15)	O4—C10—C8	110.94 (15)
O2—C4—C3	109.54 (16)	O4—C11—H11A	109.5
C5—C4—C3	112.28 (15)	O4—C11—H11B	109.5
O2—C4—C7	105.29 (13)	H11A—C11—H11B	109.5
C5—C4—C7	111.23 (16)	O4—C11—H11C	109.5
C3—C4—C7	110.48 (16)	H11A—C11—H11C	109.5
C6—C5—C4	123.44 (19)	H11B—C11—H11C	109.5
C6—C5—H5	118.3	O5—C12—H12A	109.5
C4—C5—H5	118.3	O5—C12—H12B	109.5
C5—C6—C1	121.19 (18)	H12A—C12—H12B	109.5
C5—C6—H6	119.4	O5—C12—H12C	109.5
C1—C6—H6	119.4	H12A—C12—H12C	109.5
C8—C7—C4	103.53 (14)	H12B—C12—H12C	109.5
C8—C7—H7A	111.1	O6—C13—H13A	109.5
C4—C7—H7A	111.1	O6—C13—H13B	109.5
C8—C7—H7B	111.1	H13A—C13—H13B	109.5
C4—C7—H7B	111.1	O6—C13—H13C	109.5
H7A—C7—H7B	109.0	H13A—C13—H13C	109.5
C10—C8—C7	114.59 (15)	H13B—C13—H13C	109.5
			
O1—C1—C2—C3	−174.2 (2)	C12—O5—C9—O2	55.5 (2)
C6—C1—C2—C3	7.7 (3)	C12—O5—C9—C8	−63.3 (2)
C1—C2—C3—C4	3.4 (4)	C13—O6—C9—O5	−62.06 (19)
C9—O2—C4—C5	122.48 (15)	C13—O6—C9—O2	57.0 (2)
C9—O2—C4—C3	−115.12 (16)	C13—O6—C9—C8	171.10 (15)
C9—O2—C4—C7	3.68 (19)	C4—O2—C9—O5	−151.91 (14)
C2—C3—C4—O2	−133.2 (2)	C4—O2—C9—O6	90.16 (17)
C2—C3—C4—C5	−13.5 (3)	C4—O2—C9—C8	−25.26 (18)
C2—C3—C4—C7	111.3 (2)	C10—C8—C9—O5	−79.84 (19)
O2—C4—C5—C6	134.4 (2)	C7—C8—C9—O5	157.31 (15)
C3—C4—C5—C6	13.7 (3)	C10—C8—C9—O6	40.30 (19)
C7—C4—C5—C6	−110.7 (2)	C7—C8—C9—O6	−82.55 (16)
C4—C5—C6—C1	−3.7 (3)	C10—C8—C9—O2	159.24 (14)
O1—C1—C6—C5	174.4 (2)	C7—C8—C9—O2	36.39 (17)
C2—C1—C6—C5	−7.5 (3)	C11—O4—C10—O3	1.8 (3)
O2—C4—C7—C8	19.56 (19)	C11—O4—C10—C8	−176.39 (16)
C5—C4—C7—C8	−96.87 (18)	C7—C8—C10—O3	11.3 (3)
C3—C4—C7—C8	137.73 (16)	C9—C8—C10—O3	−104.0 (2)
C4—C7—C8—C10	−154.35 (15)	C7—C8—C10—O4	−170.64 (16)
C4—C7—C8—C9	−33.39 (18)	C9—C8—C10—O4	74.07 (19)
C12—O5—C9—O6	176.6 (2)		

### Hydrogen-bond geometry (Å, º)

**Table:**

*D*—H···*A*	*D*—H	H···*A*	*D*···*A*	*D*—H···*A*
C13—H13*A*···O3^i^	0.96	2.60	3.269 (3)	127

Symmetry code: (i) −*x*+1, *y*+1/2, −*z*+1/2.
